# Traumatic hip dislocation in a child with an acetabular fleck sign indicating posterior osteochondral avulsion: a case report

**DOI:** 10.1093/jscr/rjag579

**Published:** 2026-07-13

**Authors:** Aoi Kudo, Kazuki Oishi, Seiya Ota, Masayuki Ichinohe, Yuji Yamamoto, Yasuyuki Ishibashi

**Affiliations:** Department of Orthopedic Surgery, Hirosaki University Graduate School of Medicine, 5 Zaifu-cho, Hirosaki City, Aomori 036-8562, Japan; Department of Orthopedic Surgery, Hirosaki University Graduate School of Medicine, 5 Zaifu-cho, Hirosaki City, Aomori 036-8562, Japan; Department of Orthopedic Surgery, Hirosaki University Graduate School of Medicine, 5 Zaifu-cho, Hirosaki City, Aomori 036-8562, Japan; Department of Orthopedic Surgery, Hachinohe City Hospital, 3-1-1 Tamukai, Hachinohe City, Aomori 031-8555, Japan; Department of Orthopedic Surgery, Hakodate Municipal Hospital, 1-10-1 Minato-cho, Hakodate City, Hokkaido 041-8680, Japan; Department of Orthopedic Surgery, Hirosaki University Graduate School of Medicine, 5 Zaifu-cho, Hirosaki City, Aomori 036-8562, Japan

**Keywords:** traumatic hip dislocation, pediatric hip dislocation, acetabular fleck sign, osteochondral avulsion, posterior acetabular injury

## Abstract

Pediatric traumatic hip dislocation is rare and may be accompanied by structural injuries requiring surgical treatment. We report the case of traumatic posterior hip dislocation sustained by a 9-year-old boy while playing rugby. Closed reduction was initially performed at a local hospital, but redislocation occurred 3 days later. Computed tomography demonstrated a small bony fragment adjacent to the posterior acetabular rim consistent with the acetabular fleck sign. Magnetic resonance imaging revealed an osteochondral fragment continuous with the labrum. Open reduction using the Kocher–Langenbeck approach confirmed posterior osteochondral avulsion, which was repaired with soft anchors. At the 3-year follow-up, the patient was pain-free, had a normal gait, and showed no evidence of femoral head osteonecrosis. This case suggests that the acetabular fleck sign may indicate posterior labral osteochondral avulsion in pediatric traumatic hip dislocation, and that early recognition may promote timely surgical intervention and prevent recurrent instability.

## Introduction

Traumatic hip dislocation is uncommon in children under 14 years of age, representing approximately 5% of all cases [1]. While children have a lower risk of concurrent fractures due to higher joint laxity compared with adults, delayed treatment can lead to femoral head necrosis and osteoarthritis [2]. Soft tissue interpositions, such as a torn capsule or inverted labrum, can lead to incomplete reduction and may require open reduction [3]. Imaging findings may provide important clues to such structural abnormalities. In particular, the acetabular ‘fleck sign’, a small bony fragment adjacent to the posterior acetabular rim on computed tomography (CT), is reported to indicate posterior labral osteochondral avulsion [4, 5].

We present a pediatric case of traumatic hip dislocation with an acetabular fleck sign; open reduction confirmed posterior osteochondral avulsion of the acetabular rim. This case emphasizes the diagnostic significance of this radiographic finding for timely and appropriate surgical management.

## Case presentation

A 9-year-old boy with no relevant medical or family history presented with right hip pain after sustaining a rugby-related injury. During a match, he was tackled from the left side while running and fell, supporting his body weight with his right leg. He immediately experienced right hip pain and was unable to walk. He was transported by ambulance to the referring hospital, where X-ray revealed posterior dislocation of the right hip (Fig. 1). Closed reduction was performed under general anesthesia on the day of injury. The post-treatment plan involved 4 weeks of non-weight-bearing without traction. Three days after surgery, the patient experienced increased hip pain during daily activity, and an X-ray showed redislocation of the right hip. A repeat closed reduction was performed, followed by skin traction. CT imaging revealed avulsed bone fragments (Fig. 2) and an epiphyseal defect in the posterior acetabular wall, consistent with the acetabular fleck sign. Magnetic resonance imaging (MRI) showed a detached osteochondral fragment continuous with the labrum (Fig. 3). Because structural injury was suspected, the patient was referred to our institution for surgical treatment.

**Figure 1:**
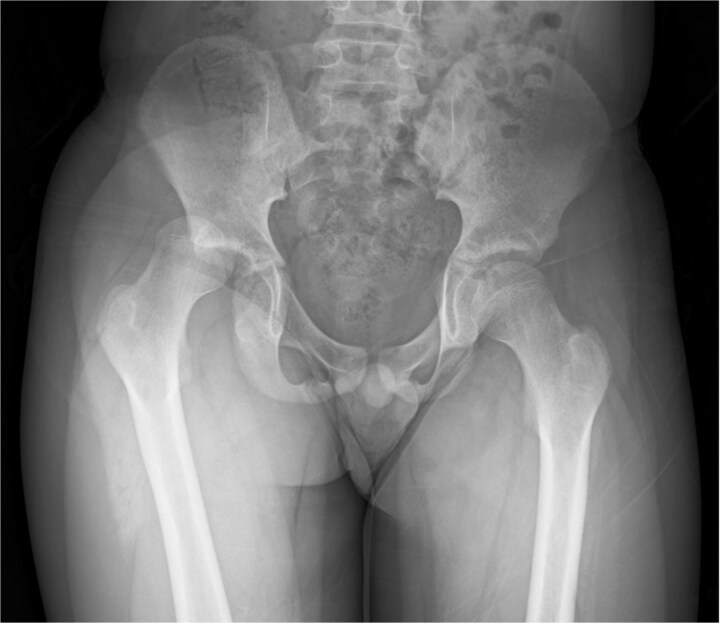
Anterior–posterior X-ray showing dislocation of the right hip.

**Figure 2:**
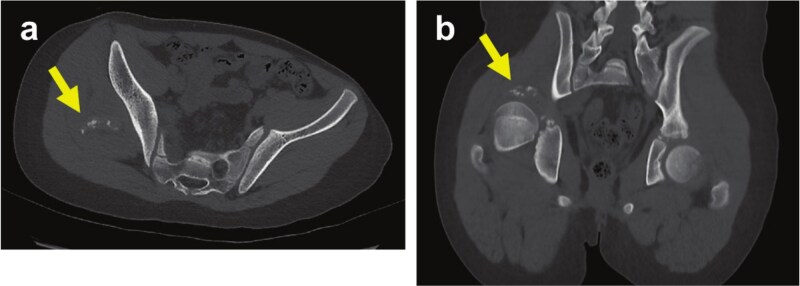
CT images of the hip before closed reduction, axial (a) and coronal (b) views. Arrows indicate an avulsed bone fragment adjacent to the posterior acetabular wall.

Five days after injury, open reduction was performed using a Kocher–Langenbeck approach with the patient in the lateral decubitus position. Intraoperative examination confirmed a posterior osteochondral fragment continuous with the acetabular labrum. The fragment was reduced and repaired using three 1.9-mm soft anchors (SUTUREFIX Ultra Anchor 1.9 mm S with two 46°ULTRABRAID sutures, Smith & Nephew, Andover, MA) and No. 1 FiberWire sutures. Postoperatively, skin traction was maintained for 2 weeks. Partial weight-bearing was started at 7 weeks, and full weight-bearing at 11 weeks. MRI at 6 months showed continued reduction without evidence of osteonecrosis (Fig. 4). At the 3-year follow-up, the patient was pain-free with a stable gait. He had returned to normal daily activities without complications.

**Figure 3:**
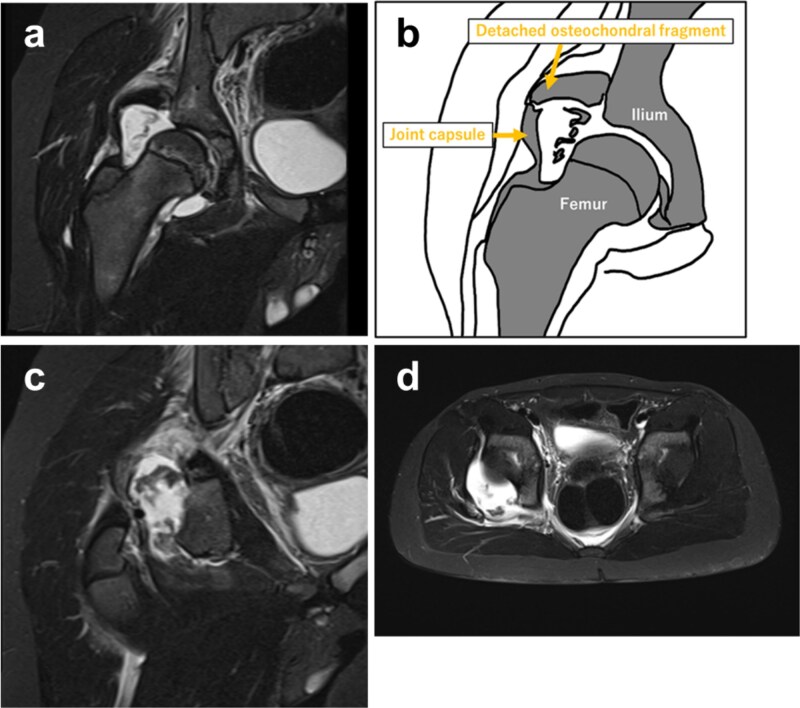
MRI on Day 3 of injury after closed reduction (a, c, d) showing an osteochondral fragment continuous with the labrum at the posterior acetabular wall.

**Figure 4:**
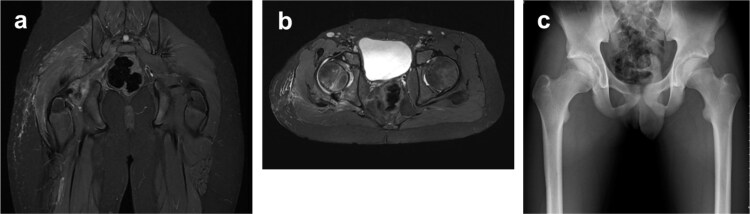
Fat-suppressed T1-weighted magnetic resonance images (a, b) on postoperative Day 17 showing maintained reduction of osteochondral fragments. X-ray (c) at 3 years postoperatively revealed no avulsed fragments.

## Discussion

This case suggests that failure to recognize the acetabular fleck sign at initial evaluation may contribute to recurrent instability following pediatric traumatic hip dislocation. In this patient, the fleck sign was not identified after the initial reduction, and redislocation occurred 3 days later. Earlier recognition of this finding may have promoted timely surgical intervention, potentially preventing recurrence.

Traumatic hip dislocation in children is uncommon but requires prompt management to avoid complications such as avascular necrosis of the femoral head [2]. Previous studies demonstrated that delaying reduction beyond 6 hours significantly increases the risk of osteonecrosis [6]. Therefore, early diagnosis and immediate reduction are essential. In addition to early reduction, identification of associated structural injuries is important. Interposed soft tissues such as the labrum, joint capsule, or osteochondral fragments may prevent concentric reduction and result in recurrent instability [2, 7]. Advanced imaging, including CT and MRI, plays a key role in evaluating these injuries [8].

The acetabular fleck sign is a radiographic marker of posterior labral osteochondral avulsion in traumatic hip dislocation [4, 5] and may indicate structural injury requiring surgical repair. In the present case, the fleck sign observed on CT corresponded to a posterior osteochondral fragment continuous with the acetabular labrum identified intraoperatively. This radiologic–surgical correlation supports the diagnostic value of the fleck sign. Earlier recognition might have prompted earlier surgical treatment and potentially prevented redislocation. Careful imaging evaluation following reduction of pediatric hip dislocation is therefore essential for identifying structural injuries that may require surgical management.

## Conclusion

The acetabular fleck sign may indicate posterior osteochondral avulsion of the acetabulum in pediatric traumatic hip dislocation. Recognition of this imaging finding may help identify cases that require surgical intervention.
